# Vestibular Complaints Impact on the Long-Term Quality of Life of Vestibular Schwannoma Patients

**DOI:** 10.1097/MAO.0000000000003773

**Published:** 2022-11-29

**Authors:** Constanza Fuentealba-Bassaletti, Olaf M. Neve, Babette F. van Esch, Jeroen C. Jansen, Radboud W. Koot, Peter Paul G. van Benthem, Erik F. Hensen

**Affiliations:** ∗ Department of Otorhinolaryngology and Head and Neck Surgery, Leiden University Medical Center; † Department of Neurosurgery, Leiden University Medical Center, Leiden, the Netherlands

**Keywords:** Active surveillance, Cerebellopontine angle tumor, Dizziness, Quality of life, Radiotherapy, Surgery, Vertigo, Vestibular schwannoma

## Abstract

To analyze the effect of dizziness-related symptoms on the long-term quality of life (QoL) of patients with unilateral vestibular schwannoma.

**Methods:**

In this cross-sectional study, patients with a unilateral vestibular schwannoma diagnosed between 2004 and 2013 completed a disease-specific QoL questionnaire (Penn Acoustic Neuroma Quality of Life [PANQOL]) and the Dizziness Handicap Inventory (DHI) in 2020. Linear regression was performed to assess the correlation between QoL and the DHI total score, and the scores of the DHI functional, emotional, and physical subdomains. Potential confounders such as age, sex, tumor size at baseline, and treatment modality (active surveillance, surgery, or radiotherapy) were included in the model.

**Results:**

In total, 287 of 479 patients (59%) experienced dizziness with a median follow-up of 10 years. The DHI total score was significantly associated with the PANQOL total score. On average, we found a reduction of 0.7 points on the PANQOL for each additional point on the DHI. The DHI emotional subdomain was the most prominent determinant of poorer QoL. Each point on the DHI emotional subscale was associated with a reduction of 1.3 on the PANQOL score. Treatment modality did not have a clinically relevant effect on dizziness-related QoL.

**Conclusions:**

Even mild dizziness can have a significant and clinically relevant effect on the QoL of patients with unilateral vestibular schwannoma in the long term. This holds true for all treatment modalities. Addressing the vestibular problems may improve QoL in vestibular schwannoma patients, and DHI subscale analysis may help tailor the optimal vestibular intervention.

## INTRODUCTION

Vestibular schwannomas are benign tumors arising from the Schwann cells of the vestibular nerve in the inner ear (1). The most common symptoms include asymmetrical sensorineural hearing loss, tinnitus, and imbalance (2). In addition to the balance compromise, vestibular schwannoma patients also frequently present with dizziness and vertigo, affecting the patient's daily activities and impacting on their quality of life (QoL) (3–5).

Current vestibular schwannoma treatment strategies can be summarized as surgery, radiotherapy, or surveillance (i.e., no active intervention). Unfortunately, all treatment modalities carry inherent risks to the vestibular function: in surgery, it is inevitably compromised because of the transection of the vestibular nerve from which the tumor arises. In radiotherapy, the tumor and its potential deleterious effect on the vestibular function are not removed, and both the vestibular organ and the vestibular nerves are exposed to radiation, which may cause additional vestibular toxicity. In active surveillance, the tumor and its impact on the vestibular function are left in situ, and the vestibular function may deteriorate as a result of the natural course of the disease. The primary aim of vestibular schwannoma therapy is therefore usually not amelioration of the audiovestibular symptoms, but prevention of future complications due to tumor progression. Even so, the reported effects of vestibular schwannoma therapy on vestibular symptoms are diverse. Some studies show no differences in vestibular symptoms of patients who underwent surgery, radiotherapy, or active surveillance, whereas others report a reduction in vestibular symptoms after translabyrinthine surgery (6–8). On the long term, a mitigation of vestibular symptoms may be expected regardless of the treatment strategy because of central compensation of unilateral vestibulopathy. However, even 7.5 years after diagnosis, half of the patients still report vestibular symptoms (6).

Several large studies have shown that vestibular symptoms are one of the most prominent determinants of QoL on vestibular schwannoma patients (3–5,9). Vestibular symptoms are more often present in female patients, older patients, and patients with larger tumors (4,10). These vestibular symptoms that vary greatly in severity are challenging to treat and have an impact on a patient's functional, physical, and emotional well-being (11,12).

Although the immediate improvement of audiovestibular symptoms or QoL does not drive the vestibular schwannoma treatment decision itself, preservation of QoL is important in vestibular schwannoma care because the tumor does not affect the life expectancy and patients generally grow old with the tumor.

The current study aims to analyze the long-term association between vestibular symptoms and disease-related QoL of vestibular schwannoma patients after active surveillance, surgery, or radiotherapy.

## METHODS

This cross-sectional survey study was part of a more extensive study on long-term vestibular schwannoma QoL outcomes (13). The study was performed at a tertiary university hospital and expert center for vestibular schwannoma. Data collection took place between June and September 2020. The local medical research and ethics committee has waived the necessity for medical ethical approval; thus, the study was approved regarding data handling and privacy regulations.

Vestibular schwannoma patients who participated in a study on QoL in 2014 were re-invited (5). This previous study included patients diagnosed and/or treated in our center between 2003 and 2014. As a result, the time interval between diagnosis and completion of the questionnaire for the current study varied between patients (median between 8 and 10 yr). Patients 18 years or older with unilateral vestibular schwannoma were included. Patients with other skull base pathologies or insufficient language proficiency to complete the questionnaires were excluded.

After providing informed consent, participants were asked to complete two questionnaires either electronically or on paper. First, a few demographic questions were asked about age, sex, and education level. Second, the Penn Acoustic Neuroma Quality of Life (PANQOL) was completed, a validated disease-specific QoL questionnaire consisting of 26 items and 7 subdomains (hearing, balance, face, anxiety, energy, pain, and general health) (14,15). The Likert scale answers were recoded to domain scales from 0 to 100, in which higher scores indicate better QoL. An overall score was calculated as the arithmetic average of the subdomains.

The minimal clinically important difference (MCID) is defined as the lowest change in patient-reported outcomes needed to reach a meaningful clinical improvement (16). The median anchored MCID for the PANQOL total score has previously been established to be 12.5 points (17).

Third, the Dizziness Handicap Inventory (DHI) was evaluated. It consists of 25 items grouped into three subdomains (functional, emotional, physical) (18–20). In this self-reported impact of dizziness assessment, patient answers were recoded to total score on a scale from 0 to 100, in which lower scores indicate less impact of dizziness. The classification of the DHI as proposed by Whitney et al. (21) was used to categorize mild (≤30), moderate (31–60), and severe (>60) handicap. The MCID of the DHI was previously established at 11 points (22). In total, 255 patients received the questionnaires electronically. In these cases, the DHI was conditionally deployed. When patients scored at least 4 points at one or more of the PANQOL dizziness subdomain questions, they were asked to complete the DHI. All patients completing the paper questionnaire were asked to complete the DHI because the conditional aspect could not be incorporated in the paper version.

Tumor size and treatment modality were acquired from the patient records. Treatment modality was categorized as active surveillance, surgery, radiotherapy, or both surgery and radiotherapy. Patients receiving both surgery and radiotherapy did so because the initial treatment did not result in adequate tumor control, that is, the second modality (either surgery or radiotherapy) was performed as salvage treatment. Tumor size was scored at diagnosis using the reporting system proposed by Kanzaki et al. (23). The definition of Statistics Netherlands (CBS) for low, middle, and high education levels were used, following the international standard classification of education (23). Statistical analyses were performed in R version 4.1.1 using Rstudio 1.4.1717 (Rstudio; PBC, Boston, MA) and data plotted using the package *ggplot2*. Means and standard deviation were calculated for numerical variables, and when not normally distributed, medians and interquartile ranges (IQRs) were used. Frequencies and percentages were calculated in the case of categorical variables. DHI score categories were compared per treatment modality using *χ*^2^ test with Bonferroni correction for multiple testing. DHI scores were compared using the Wilcoxon rank test. Linear regression was performed to assess the effect of DHI scores on QoL (dependent variable). Potential confounders such as age, sex, tumor size at treatment, time since the treatment, and treatment modality were included in the model based on previous research (4,5). Furthermore, the determinants of dizziness were explored in a linear model with DHI total score as the dependent variable. Model assumptions were checked visually. The effect of the DHI subdomains (functional, emotional, and physical) on QoL was assessed in an additional regression analysis, including the potential confounders mentioned previously. A *p* value <0.05 was considered statistically significant.

## RESULTS

**FIG. 1:**
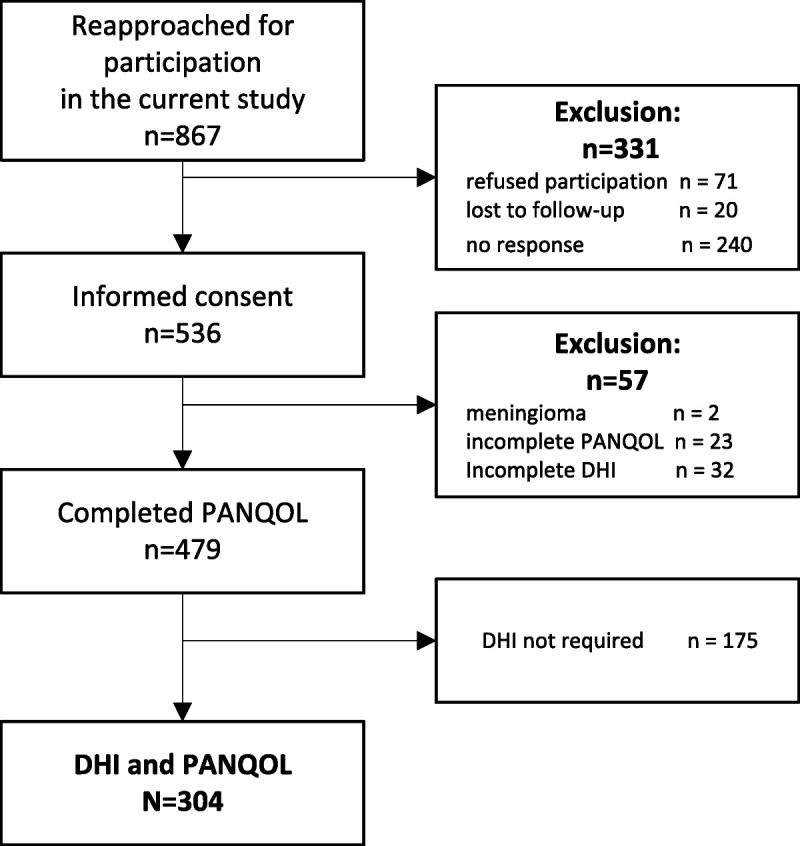
Flowchart of study participants.

As shown in Figure 1, 536 of 867 patients (62%) responded and provided informed consent. Compared with nonresponders, responders were, on average, 9 months younger, had higher education levels (high-level education, 21% versus 14%), and had received surgery more often (22% versus 10%). Two patients were excluded because of different definitive diagnoses (meningioma instead of vestibular schwannoma in both cases), and 55 were excluded because they failed to complete either the PANQOL or DHI questionnaire.

In all, 479 of 865 vestibular schwannoma patients (55%) completed at least one of the questionnaires (PANQOL and/or DHI), of whom 41% did not experience any vestibular symptoms, 37% had mild symptoms, 18% had moderate symptoms, and 4% had severe symptoms. Patients who underwent active treatment suffered more often from vestibular symptoms (68% of surgical patients and 73% of radiotherapy patients) compared with patients who remained under active surveillance (49%). The overall PANQOL score of patients with dizziness was significantly worse compared with patients without vestibular symptoms (Δ21.5, *p* < 0.001); this difference exceeded the MCID of 12.5 points and thus is deemed clinically relevant. Furthermore, patients who scored positive on the PANQOL balance subdomain questions had a significantly higher DHI score (Δ20 points, *p* < 0.001) than patients who did not, supporting the conditional deployment of the DHI questionnaire.

The questionnaires were completed on paper by 49 patients and electronically by 255 patients. In the latter group, the DHI was deployed conditionally, and 175 patients did not complete the DHI because of their (low) PANQOL balance domain score. Both DHI and PANQOL questionnaires were completed by 304 patients; their baseline characteristics are shown in Table 1. The median time after diagnosis was 10 years (IQR, 8–13 yr), and the median time since active treatment was 9 years (IQR, 8–12 yr). The mean age was 67.7 years, and 55.4% of the participants was female.

**TABLE 1:** Baseline characteristics

		Treatment Modality			
	Total	Active Surveillance	Surgery	Radiotherapy	Salvage Therapy
N	304	127	122	42	13
Age, mean (SD), yr	67.7 (10.8)	70.1 (10.5)	65.0 (10.1)	69.3 (11.1)	64.1 (14.2)
Women, n (%)	170 (55.4)	64 (50.4)	77 (61.6)	22 (52.4)	7 (53.8)
Education, n (%)					
Low	112 (36.7)	58 (45.7)	37 (30.1)	13 (31.0)	4 (30.8)
Middle	90 (29.5)	30 (23.6)	44 (35.8)	11 (26.2)	5 (38.5)
High	103 (33.8)	39 (30.7)	42 (34.1)	18 (42.9)	4 (30.8)
Time since, yr					
Treatment, median (IQR)	9 (8–12)	—	10 (8–13)	8 (5–10)	9 (8–11)
Diagnosis, median (IQR)	10 (8–13)	9 (8–11)	11 (9–14)	10 (8–12)	11 (9–13)
Kanzaki at treatment*^a^*, n (%)					
Intrameatal	113 (36.7)	71 (55.9)	28 (22.4)	13 (31.0)	1 (7.7)
Small (0–10 mm)	63 (20.5)	26 (20.5)	21 (16.5)	11 (26.2)	5 (38.5)
Medium (11–20 mm)	72 (23.3)	24 (18.9)	32 (25.2)	12 (28.6)	4 (30.8)
Moderately large (21–30 mm)	32 (10.4)	3 (2.3)	24 (18.9)	4 (9.5)	1 (7.7)
Large + giant (>30 mm)	22 (7.1)	1 (<1)	19 (14.9)	0	2 (15.4)
Missing	7	2	3	2	0

*^a^*For active surveillance, tumor size in 2014 was used.

IQR, interquartile range; SD, standard deviation; —, active surveillance time since treatment = time since diagnosis.

**FIG. 2:**
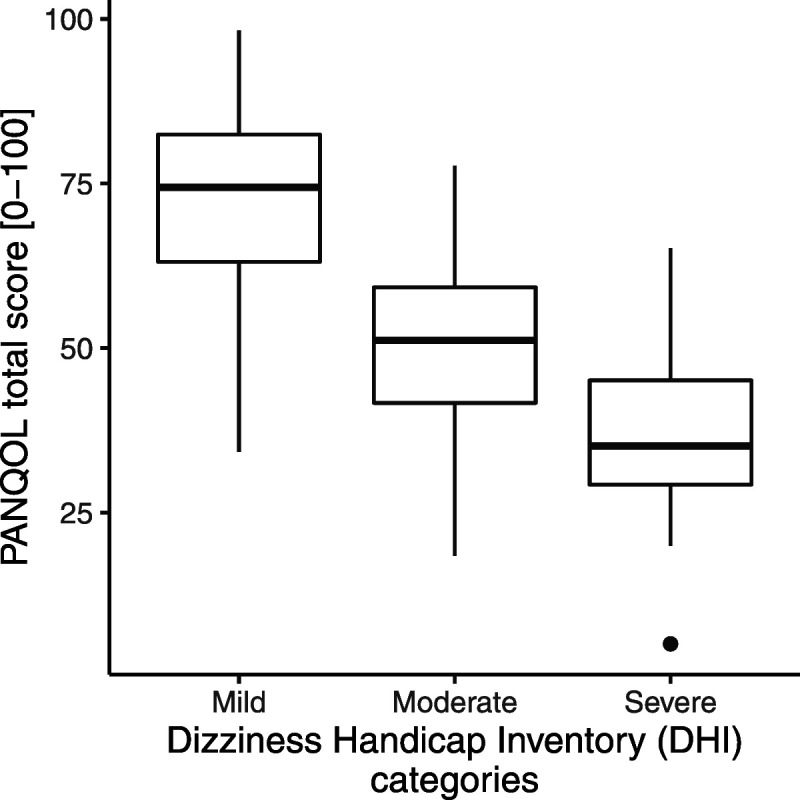
DHI severity categories related to QoL total scores. From the 304 patients who completed both the PANQOL and the DHI, the patients with severe complaints (36.5 ± 13.5) have significant worse QoL scores than patients with mild vestibular complaints (72.2 ± 13.3). DHI indicates Dizziness Handicap Inventory; QoL, quality of life; PANQOL, Penn Acoustic Neuroma Quality of Life.

**FIG. 3:**
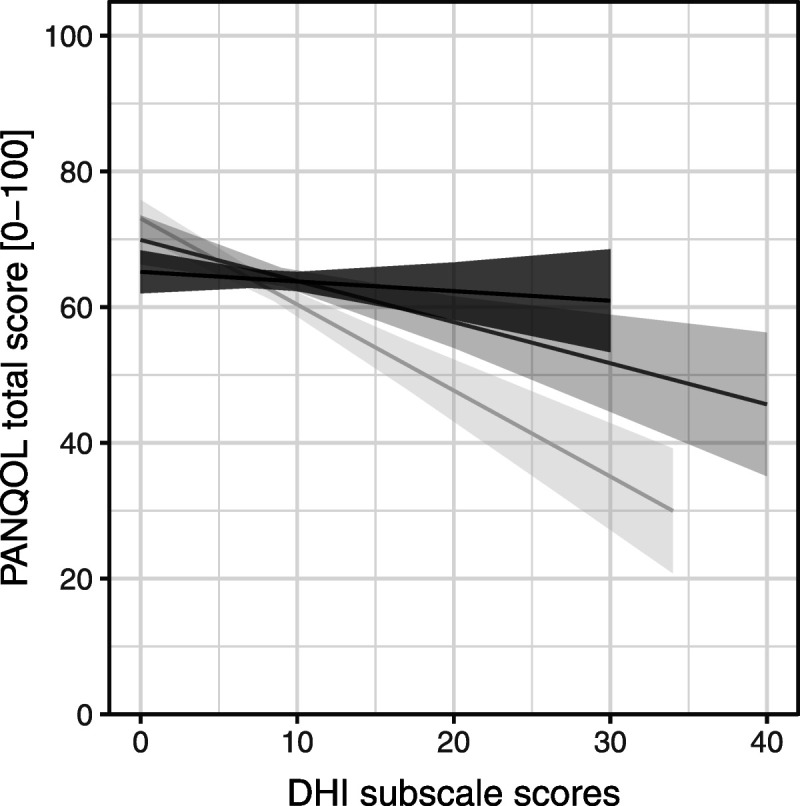
DHI subscales scores related to PANQOL total scores. The emotional subscale has the strongest association with QoL (−1.3; 95% CI, −1.6 to −1.0) compared with the functional (−0.6; 95% CI, −0.9 to −0.3) and the physical subscale (−0.1; 95% CI, −0.4 to 0.2). *Light gray line* represents emotional subscale; *gray line*, functional subscale; and *black line*, physical subscale. CI indicates confidence interval; DHI, Dizziness Handicap Inventory; QoL, quality of life; PANQOL, Penn Acoustic Neuroma Quality of Life.

The association between DHI and disease-specific QoL scores could only be evaluated in patients who completed both the PANQOL and DHI questionnaire (n = 304). There was a significant association between DHI total score and PANQOL total score after correction for confounding factors. Mean PANQOL total score of patients with a severe dizziness handicap (mean ± standard deviation, 36.5 ± 13.5) was lower compared with moderate (51.7 ± 12.4) and mild dizziness (72.2 ± 13.3; Fig. 2). These differences were statistically significant and clinically relevant because they exceeded the PANQOL MCID. For every incremental DHI point, the score of the PANQOL deteriorated with 0.7 points (95% confidence interval [CI], −0.7 to −0.6). When the DHI subscales were analyzed separately, the emotional subscale seemed to have the strongest association with QoL (−1.3; 95% CI, −1.6 to −1.0), whereas the physical subscale was not associated with PANQOL total score (−0.1; 95% CI, −0.4 to 0.2; Fig. 3). The MCID of the PANQOL (12.5 points) was exceeded when the DHI total score exceeded 18 points and/or when the DHI emotional subscale score exceeded 9 points. From the 304 patients who completed the DHI, 180 patients (59%) had a DHI total score of at least 18 points (60 under active surveillance, 85 after surgery, 26 after radiotherapy, and 9 after salvage therapy), and 104 patients (34%) scored at least 9 in the DHI emotional subdomain (32 under active surveillance patients, 52 after surgical patients, 17 after radiotherapy patients, and 3 after salvage therapy patients).

The determinants of DHI score are shown in Table 2. Age, education level, tumor size, and time since the treatment did not influence DHI total score. Female sex seemed to be a determinant for an increased impact of vestibular symptoms. After correction for potentially confounding factors, female participants, on average, scored 9.1 points (95% CI, 4.5 to 13.7) higher than male participants on the DHI, a statistically significant difference. Patients who underwent surgery had significantly higher DHI scores (8.2; 95% CI, 1.1 to 15.3) than patients who remained under active surveillance. Patients who underwent radiotherapy (4.3; 95% CI, −4.9 to 14.3) or salvage therapy (10.2, 95% CI, −4.7 to 25.0) also had higher DHI scores; however, these differences were not statistically significant, which in the latter group might be caused by the small sample size. None of the associations mentioned previously exceed the MCID of 11 points, and thus, none are deemed clinically relevant; however, the combination of factors can exceed the MCID. For example, female participants who underwent surgery scored 17.2 points higher on the DHI compared with male participants who underwent active surveillance. There were no significant interaction terms identified, especially the association between treatment and DHI score, which was not dependent on the time since treatment.

**TABLE 2:** Liner regression analysis shows the determinants for the Dizziness Handicap Inventory score

	Dependent Variable Total DHI			
	Estimated Mean Total DHI	95% CI	Marginal Means	95% CI
Female	30.0	26.0 to 34.0	—	
Male	20.9	16.5 to 25.3	**−9.1**	**−13.7 to −4.5**
Age	25.4	21.9 to 29.0	0.0	−0.2 to 0.2
Time since the treatment	25.5	21:9 to 29.0	0.2	−0.5 to 0.9
Education level				
Low	23.6	18.9 to 28.3	—	
Middle	27.9	22.9 to 32.9	4.3	−2.5 to 11.0
High	24.9	20.2 to 29.5	1.3	−5.4 to 8.0
Treatment				
Active surveillance	19.6	15.3 to 24.0	—	
Surgery	27.8	24.3 to 31.4	**8.2**	**1.1 to 15.3**
Radiotherapy	24.5	18.0 to 31.	4.3	−4.9 to 14.3
Salvage therapy	29.8	19.8 to 40.3	10.2	−4.7 to 25.0
Kanzaki				
Intrameatal	27.4	22.7 to 32.1	—	
Small (<11 mm)	29.6	24.4 to 34.8	2.2	−6.3 to 10
Medium (11–20 mm)	25.8	20.8 to 30.8	−1.6	−9.9 to 6.7
Moderately large (21–30 mm)	20.1	12.6 to 27.5	−7.4	−18.7 to 4.0
Large + giant (>30 mm)	24.4	15.6 to 33.1	−3.1	−16.4 to 10.3
Observations	291			
*χ* ^2^	<0.001			

Significant differences are shown in bold.

CI indicates confidence interval; DHI, Dizziness Handicap Inventory.

## DISCUSSION

This cross-sectional study shows that, on the long-term, a significant proportion of vestibular schwannoma patients suffer from ongoing vestibular complaints. This holds true for all treatment strategies (radiotherapy, surgery, and active surveillance). After a median follow-up time of 10 years, 59% of the vestibular schwannoma patients suffered from vestibular complaints to different degrees, and 22% of the respondents reported moderate to severe vestibular complaints. When correlating QoL (as assessed by the PANQOL questionnaire) and dizziness (evaluated with the DHI), we found that patients who reported more vestibular symptoms reported significantly lower QoL than patients without. Moreover, patients with moderate or severe vestibular complaints had significantly lower QoL scores than patients with mild complaints. These differences were not only statistically significant but also clinically relevant. The finding that vestibular complaints have a major impact on QoL is in line with previous reports (5,6,9,24,25). The current study shows that vestibular symptoms affect the QoL of vestibular schwannoma patients on the long term and may do so regardless of the chosen treatment modality. The strong correlation between QoL and vestibular complaints is illustrated by the observation that, in the current study, the PANQOL total score drops 0.7 points on average for every incremental point on the DHI total score. This means that for a DHI total score of 18 points or more, on average, there is a clinically relevant impact on the PANQOL total score. In other words, even mild vestibular complaints (defined as a DHI score of 30 points or less) may have a considerable effect on the long-term QoL of vestibular schwannoma patients.

A possible determinant of vestibular complaints is the treatment modality. Patients who underwent radiotherapy, surgery, or both scored higher on the DHI than patients who underwent active surveillance. However, these differences did not exceed the DHI MCID of 11 points. Treatment modality therefore does not seem to have a clinically relevant differential effect on vestibular complaints and QoL on the long term.

The question that arises is why so many patients are still suffering from vestibular complaints long after vestibular schwannoma diagnosis and/or treatment. It is generally believed that central compensation of unilateral vestibular function loss should take place in the majority of patients, diminishing their symptoms over time (26). Our findings suggest that vestibular compensation of unilateral vestibulopathy is not always satisfactory in vestibular schwannoma patients and that spontaneous vestibular recovery does not always take place. There is good evidence that vestibular rehabilitation exercises have a beneficial effect on the functional outcome after unilateral vestibular function loss in vestibular schwannoma patients, and vestibular exercises are therefore commonly advised for patients suffering from vestibular complaints. There is also some evidence that vestibular “prehabilitation,” that is, vestibular exercises combined with intratympanic gentamicin treatment before vestibular schwannoma therapy may have a beneficial effect on posttherapy vestibular outcome (27). However, the long-term benefits of prehabilitation are still unclear. Moreover, both vestibular rehabilitation and prehabilitation strategies mainly address the functional and physical aspects of vestibular function loss and may fail to address its emotional impact (28,29). Because this latter aspect seems to have the strongest correlation with a decrease in QoL and seems to play a role in a considerable proportion of vestibular schwannoma patients (34%), vestibular exercises only may not be the optimal strategy. In addition to conventional vestibular rehabilitation, counseling targeted at the emotional burden may help improve the QoL in a considerable proportion of vestibular schwannoma patients. Analyzing the DHI subdomains separately may help identify the patients that would benefit from this approach, as a score of at least 9 points on the emotional DHI subscale is associated with a clinically relevant decrease in QoL.

Interestingly, we found that female sex seems to be a determinant for significantly increased score on the DHI in vestibular schwannoma patients (although the difference between men and women did not exceed the MCID in the current study) (6,30,31). This finding agrees with a report by Carlson et al. (6) A possible explanation for this observation offered by Carlson et al. is that other factors associated with female sex aggravate the dizziness-related symptoms, such as migraine and anxiety, and that the higher DHI scores in women are therefore not due to the presence of the vestibular schwannoma per se.

Some important strengths and limitations apply to the current study. First, we were able to include an adequate sample size with a good representation of vestibular schwannoma patients with diverse clinical presentations and management strategies. Second, the average duration of 10 years between diagnosis and vestibular survey helps to improve our understanding of the importance and impact of vestibular complaints on QoL on the long term. However, the study design does not allow for identification of pretreatment patient characteristics that might indicate which vestibular schwannoma treatment modality would best resolve the individual patients' vestibular complaints.

Last, with a single questionnaire such as the DHI, it may be hard to capture the full complexity of the emotional burden of dizziness, which involves experiential, behavioral, and psychological elements; however, the DHI can be used as screening tool to explore the emotional dimension of the vestibular symptoms (32,33).

## CONCLUSION

Most patients with unilateral vestibular schwannoma suffer from dizziness in the long-term (59%). Even mild vestibular complaints may have a clinically relevant impact on QoL.

The DHI may be an important instrument to explore the different dimensions of the vestibular complaints in vestibular schwannoma patients and help tailor the optimal vestibular intervention. Because the emotional burden of dizziness is associated with a clinically relevant reduction in QoL in a considerable proportion of patients (34%), addressing this aspect specifically may be of great value in improving long-term QoL in vestibular schwannoma patients.

## References

[1] Rosahl S Bohr C Lell M Hamm K Iro H. Diagnostics and therapy of vestibular schwannomas—An interdisciplinary challenge. *GMS Curr Top Otorhinolaryngol Head Neck Surg* 2017;16:Doc03. 29279723 10.3205/cto000142 PMC5738934

[2] Rosenberg SI. Natural history of acoustic neuromas. *Laryngoscope* 2000;110:497–508. 10763994 10.1097/00005537-200004000-00002

[3] Jufas N Flanagan S Biggs N Chang P Fagan P. Quality of life in vestibular schwannoma patients managed by surgical or conservative approaches. *Otol Neurotol* 2015;36:1245–54. 26075673 10.1097/MAO.0000000000000789

[4] Carlson ML Tveiten ØV Driscoll CL, . What drives quality of life in patients with sporadic vestibular schwannoma? *Laryngoscope* 2015;125:1697–702. 25546382 10.1002/lary.25110

[5] Soulier G van Leeuwen BM Putter H, . Quality of life in 807 Patienst with vestibular schwannoma: comparing treatment modalities. *Otolaryngol Head Neck Surg* 2017;157:92–8. 28319458 10.1177/0194599817695800

[6] Carlson ML Tveiten ØV Driscoll CL, . Long-term dizziness handicap in patients with vestibular schwannoma: a multicenter cross-sectional study. *Otolaryngol Head Neck Surg* 2014;151:1028–37. 25273693 10.1177/0194599814551132

[7] Carlson ML Barnes JH Nassiri A, . Prospective study of disease-specific quality-of-life in sporadic vestibular schwannoma comparing observation, radiosurgery, and microsurgery. *Otol Neurotol* 2021;42:199–208. 10.1097/MAO.0000000000002863 33177408

[8] Godefroy WP Hastan D van der Mey AGL. Translabyrinthine surgery for disabling vertigo in vestibular schwannoma patients. *Clin Otolaryngol* 2007;32:167–72. 17550503 10.1111/j.1365-2273.2007.01427.x

[9] Myrseth E Møller P Wentzel-Larsen T Goplen F Lund-Johansen M. Untreated vestibular schwannoma: Vertigo is a powerful predictor for health-related quality of life. *Neurosurgery* 2006;58:67–76. 10.1227/01.neu.0000243285.06415.4c 28180608

[10] Harun A Agrawal Y Tan M Niparko JK Francis HW. Sex and age associations with vestibular schwannoma size and presenting symptoms. *Otol Neurotol* 2012;33:1604–10. 22996162 10.1097/MAO.0b013e31826dba9e

[11] Kjærsgaard JB Szeremet M Hougaard DD. Vestibular deficits correlating to dizziness handicap inventory score, hearing loss, and tumor size in a Danish cohort of vestibular schwannoma patients. *Otol Neurotol* 2019;40:813–9. 31135674 10.1097/MAO.0000000000002236

[12] Brown CS Cooper MW Peskoe SB Risoli T Jr. Kaylie DM. Associations of vestibular tests with Penn Acoustic Neuroma Quality of Life scores after resection of vestibular schwannoma. *Otol Neurotol* 2020;41:e241–9. 31821250 10.1097/MAO.0000000000002462

[13] Neve OM Jansen JC Koot RW, . Long-term quality of life of vestibular schwannoma patients: A longitudinal analysis. *Otolaryngol Head Neck Surg* 2022;1945998221088565. 35349360 10.1177/01945998221088565

[14] Shaffer BT Cohen MS Bigelow DC Ruckenstein MJ. Validation of a disease-specific quality-of-life instrument for acoustic neuroma: The Penn Acoustic Neuroma Quality-of-Life scale. *Laryngoscope* 2010;120:1646–54. 20641085 10.1002/lary.20988

[15] van Leeuwen BM Herruer JM Putter H, . Validating the Penn Acoustic Neuroma Quality of Life scale in a sample of Dutch patients recently diagnosed with vestibular schwannoma. *Otol Neurotol* 2013;34:952–7. 23714709 10.1097/MAO.0b013e31828bb2bb

[16] Jaeschke R Singer J Guyatt GH. Measurement of health status. Ascertaining the minimal clinically important difference. *Control Clin Trials* 1989;10:407–15. 2691207 10.1016/0197-2456(89)90005-6

[17] Kerezoudis P Yost KJ Tombers NM, . Defining the minimal clinically important difference for patients with vestibular schwannoma: Are all quality-of-life scores significant? *Neurosurgery* 2019;85:779–85. 30395303 10.1093/neuros/nyy467

[18] Jacobson GP Newman CW. The development of the Dizziness Handicap Inventory. *Arch Otolaryngol Head Neck Surg* 1990;116:424–7. 2317323 10.1001/archotol.1990.01870040046011

[19] Vereeck L Truijen S Wuyts FL Van de Heyning P. The Dizziness Handicap Inventory and its relationship with functional balance performance. *Otol Neurotol* 2007;28:87–93. 17195749 10.1097/01.mao.0000247821.98398.0d

[20] Vereeck L Truijen S Wuyts F Van de Heyning P. Test-retest reliability of the Dutch version of the Dizziness Handicap Inventory. *B-ENT* 2006;2:75–80. 16910291

[21] Whitney SL Wrisley DM Brown KE Furman JM. Is perception of handicap related to functional performance in persons with vestibular dysfunction? *Otol Neurotol* 2004;25:139–43. 15021773 10.1097/00129492-200403000-00010

[22] Tamber AL Wilhelmsen KT Strand LI. Measurement properties of the Dizziness Handicap Inventory by cross-sectional and longitudinal designs. *Health Qual Life Outcomes* 2009;7:101. 20025754 10.1186/1477-7525-7-101 PMC2804706

[23] Kanzaki J Tos M Sanna M Moffat DA. New and modified reporting systems from the consensus meeting on systems for reporting results in vestibular schwannoma. *Otol Neurotol* 2003;24:642–9. 12851559 10.1097/00129492-200307000-00019

[24] Hirvonen M Aalto H Petteri Hirvonen T. Motorized head impulse rotator in patients with vestibular schwannoma. *Acta Otolaryngol* 2008;128:1215–20. 18607973 10.1080/00016480801908027

[25] Pruijn IMJ Kievit W Hentschel MA Mulder JJS Kunst HPM. What determines quality of life in patients with vestibular schwannoma? *Clin Otolaryngol* 2020;46:412–20. 33326685 10.1111/coa.13691 PMC7986908

[26] Lacour M Helmchen C Vidal PP. Vestibular compensation: the neuro-otologist's best friend. *J Neurol* 2016;263(Suppl 1):S54–64. 27083885 10.1007/s00415-015-7903-4 PMC4833803

[27] Magnusson M Kahlon B Karlberg M Lindberg S Siesjo P. Preoperative vestibular ablation with gentamicin and vestibular ‘prehab’ enhance postoperative recovery after surgery for pontine angle tumours—First report. *Acta Otolaryngol* 2007;127:1236–40. 17917842 10.1080/00016480701663433

[28] Cowand JL Wrisley DM Walker M Strasnick B Jacobson JT. Efficacy of vestibular rehabilitation. *Otolaryngol Head Neck Surg* 1998;118:49–54. 9450828 10.1016/S0194-5998(98)70374-2

[29] Hrubá S Chovanec M Čada Z, . The evaluation of vestibular compensation by vestibular rehabilitation and prehabilitation in short-term postsurgical period in patients following surgical treatment of vestibular schwannoma. *Eur Arch Otorhinolaryngol* 2019;276:2681–9. 31187238 10.1007/s00405-019-05503-8

[30] Driscoll CL Lynn SG Harner SG Beatty CW Atkinson EJ. Preoperative identification of patients at risk of developing persistent dysequilibrium after acoustic neuroma removal. *Am J Otol* 1998;19:491–5. 9661760

[31] Feigl GC Schebesch KM Rochon J, . Analysis of risk factors influencing the development of severe dizziness in patients with vestibular schwannomas in the immediate postoperative phase. *Clin Neurol Neurosurg* 2011;113:52–6. 20965648 10.1016/j.clineuro.2010.09.002

[32] Duracinsky M Mosnier I Bouccara D Sterkers O Chassany O. Literature review of questionnaires assessing vertigo and dizziness, and their impact on patients' quality of life. *Value Health* 2007;10:273–84. 17645682 10.1111/j.1524-4733.2007.00182.x

[33] Association American Psychological. APA Dictionary of Psychology. Available at: https://dictionary.apa.org/emotion. Accessed June 23, 2022.

